# Effect of Corn Origin on Broiler Performance, Processing Yield, and Nutrient Digestibility from 1 to 35 Days of Age

**DOI:** 10.3390/ani13071248

**Published:** 2023-04-04

**Authors:** Jose I. Vargas, Joseph P. Gulizia, Susan M. Bonilla, Santiago Sasia, Wilmer J. Pacheco

**Affiliations:** Department of Poultry Science, Auburn University, Auburn, AL 36849, USA

**Keywords:** corn origin, broilers, growth performance, processing yield, nutrient digestibility

## Abstract

**Simple Summary:**

Understanding the physical, chemical, and nutritional variability of corn and its impact on broiler performance is crucial for poultry nutritionists and feed mills that use corn of different origins. In the present study, broilers were fed commercial diets formulated to only differ in corn origin (United States, Argentina, and Brazil) to assess its impact on growth performance, carcass traits, and nutrient digestibility. The results indicated the impact of corn origin on feed efficiency and breast weight. Likewise, corn origin had an impact on mineral digestibility.

**Abstract:**

This experiment studied the effect of feeding corn from the United States (USA), Argentina (ARG), and Brazil (BRA) on broiler performance, processing yield, and nutrient digestibility from 1 to 35 d of age. A total of 900-day old YPM × Ross 708 male broilers were randomly sorted in 36 floor pens, each containing 25 chicks and subjected to three experimental diets, resulting in 12 replications per dietary treatment. Starter, grower, and finisher diets were formulated to be only different in corn origin. Data were analyzed as a one-way ANOVA and means were separated using Tukey’s HSD test, with statistical significance considered at *p* ≤ 0.05. No significant differences (*p* > 0.05) were found in body weight, body weight gain, and feed intake from 1 to 35 d of age among the treatments. However, broilers fed diets with the inclusion of corn from the USA and BRA had a lower feed conversion ratio (FCR) compared with broilers fed diets with corn from ARG from to 1 to 35 d of age (1.434 and 1.434 vs. 1.452 g:g; *p* = 0.002). Broilers fed diets with the inclusion of corn from BRA had a higher breast weight compared with broilers fed diets with the inclusion of corn from ARG (575 vs. 553 g; *p* = 0.036), but did not differ in breast weight of broilers fed diets with corn from the USA (575 vs. 556 g; *p* > 0.05). Corn origin did not influence (*p* > 0.05) crude protein and fat digestibility. However, broilers fed diets with corn from the USA and BRA had greater phosphorus (P) (63.37, 62.23 vs. 55.26%; *p* = 0.0003), calcium (Ca) (41.59, 43.85 vs. 30.23%; *p* = 0.0003), and potassium (K) (88.98, 87.97 vs. 86.04%; *p* = 0.001) digestibility compared with broilers fed diets with corn from ARG. Overall, corn origin influenced FCR, breast weight, and P, Ca, and K digestibility of broilers from 1 to 35 d of age.

## 1. Introduction

Worldwide, corn is used in poultry feedstuffs due to its high content of available energy [1,2]. Corn can contribute around 65% of the apparent metabolizable energy (AME) and 20% of the crude protein (CP) of a broiler diet [3]. The nutritional value of corn in broiler diets is determined by its chemical composition [3,4] and physical characteristics [5,6]. Nonetheless, its content of starch (67–76% dry matter) [7], protein (7.9–11.7% dry matter) [7], oil (3.7–7.5% dry matter) [8], amino acids (AA) (Lys: 2.98–4.89% CP air-dry basis; Met: 0.83–1.64% CP air-dry basis) [9], and non-starch polysaccharides (55.6–81.3 g/kg as is) [6,10] is variable and influenced by genetic background [11,12], growing conditions, post-harvest processing [5,13,14], and storage conditions [15]. In addition, variability in physical characteristics of corn, such as hardness (34.9–80.0%) [16,17] and kernel density (60–71 kg/hL) [18,19], are associated with its genetic background [20], agronomic practices, post-harvest processing, and drying temperatures [18,21].

Knowledge of the nutritional value of corn is critical for feed mills using corn of different origins throughout the year due to changes in suppliers caused by shifts in seasonality and price. In this context, the formulation of diets using average nutritional values assumes consistency among corn batches [11,22], and could impact broiler performance and processing yield. Therefore, understanding the influence of origin on corn nutritional variability is necessary for poultry nutritionists to formulate diets more precisely [23] and meet performance objectives.

The objective of this study was to determine the effect of the dietary inclusion of corn samples of different origins: United States (USA), Argentina (ARG), and Brazil (BRA) on growth performance, nutrient digestibility, and processing yield of broilers from 1 to 35 d of age.

## 2. Materials and Methods

### 2.1. Animal Care

The poultry experiment reported herein was reviewed and approved by the Auburn University Institutional Care and Use Committee (PRN 2021-3874).

### 2.2. Bird Husbandry

A total of 900 male broiler chicks were obtained from a parent stock flock, YPM × Ross 708 (Aviagen North America, Huntsville, AL, USA), and placed in a solid-sided house equipped with a negative pressure ventilation system, inlets, exhaust fans, evaporative cooling system, forced-air heaters, and electronic control to manage temperature. Chicks were randomly sorted into 36 floor pens (25 birds/pen; 0.12 m^2^/bird) equipped with 5 nipple drinkers, a hanging pan feeder, and fresh litter, to obtain 12 replications per treatment. Feed and water were provided ad libitum throughout the experimental period. The photoperiod was set at 23L:1D, with an intensity of 4.0-foot candles (43 lux) from 1 to 7 d of age, 21L:3D and 1.0-foot candle (11 lux) from 8 to 10 d of age, and 20L:4D and 0.50-foot candles (5 lux) from 11 to 35 d of age. Environmental temperature was set at 33 °C at placement and was gradually reduced to 20 °C by 35 d of age. Bird comfort, mortality, temperature, humidity, and availability of feed and water were inspected twice a day. Body weight gain (BWG), body weight (BW), feed intake (FI), and mortality-adjusted feed conversion ratio (FCR) were determined at 10, 21 and 35 d of age.

### 2.3. Feed Formulation, Manufacture, and Experimental Design

Whole corn from the USA (re-imported), ARG, and BRA was received from Cartagena, Colombia (gathering point), for the elaboration of the experimental diets described herein. A representative compound sample from each origin was collected and sent to a federally designated laboratory (North Dakota Grain Inspection Service, Fargo, ND, USA) to be analyzed for test weight, damaged kernels, broken corn and foreign material (BCFM), and total stress cracks. All corn was ground to a target particle size of 600 μm using a hammer mill (Bliss, Ponka, OK, USA) equipped with a 92.52 cm rotor, 50 hp motor, and 4.8 and 5.6 mm full-circle screens. Three sets of 5 representative ground corn samples were collected from each origin. The first set was used for particle size analysis using a Dura Tap shaker (Advantech, Mentor, OH, USA). The geometric mean diameter of particles was determined based on the ASABE [24] method S319.4. The second set was sent to a commercial laboratory (Dairyland, Arcadia, WI, USA) to be nutritionally characterized using near-infrared reflectance spectroscopy with a Phoenix 5000 spectrometer (BlueSun Scientific Inc., Jessup, MD, USA). The third set was sent to the Agricultural Experiment Station Chemical Laboratories of the University of Missouri-Columbia for AA profile determination using high-performance liquid chromatography (method 982.30 E (a,b,c), chp. 45.3.05 and method 988.15, chp. 45.4.04; AOAC International, [25]).

All diets were manufactured at the Auburn University Feed Mill at the Charles C. Miller Research and Education Center. Ingredients were blended for 150 s (30 s dry cycle and 120 s wet cycle) using a twin shaft mixer (Model 726, Scott Equipment Co., New Prague, MN, USA) to produce the mash diets. Mash diets were conditioned at 79 °C for 40 s and pelleted through a 4.0 mm pellet die using a pellet mill (Model 1112-4, California Pellet Mill Co., Crawfordsville, IN, USA). Pellets were cooled with ambient air using a counter-flow pellet cooler (Model CC0909, California Pellet Mill Co., Crawfordsville, IN, USA). Starter diets were crumbled in a crumbler with manual roll adjustment (Model 624SS, California Pellet Mill Co., Crawfordsville, IN, USA).

The experimental treatments consisted of starter, grower, and finisher diets containing the same % of each corn origin, formulated to fulfill Aviagen recommendations for Ross 708 male broilers [26] (Table 1). All diets had an inclusion of 500 phytase units (FTU)/kg of OptiPhos^®^ Plus (heat stable commercial phytase), which have an activity of 10,000 FTU/g (Huvepharma Inc., Peachtree City, GA, USA). According to AOAC [27], a single FTU is equivalent to the amount of enzyme that releases 1 micromole of inorganic orthophosphate from a sodium phytate substrate per minute at pH 5.5 and 37 °C. Therefore, the only difference between diets within a growing period was the corn used. Each pen was randomly assigned to 1 of the 3 experimental diets, obtaining 12 replicate pens per treatment. Birds were subjected to dietary treatments from 1 to 35 d of age. Broilers were provided 0.363 kg of starter feed (crumbles) from 1 to 10 d, 0.908 kg of grower (pellets) from 11 to 21 d, and ad libitum finisher (pellets) from 21 to 35 d of age.

### 2.4. Measurements

The weights of birds and feed were determined at placement, 10, 21, and 35 d of age, to calculate BW, FI, and FCR. Birds were inspected twice a day to ensure their comfort, adequate room temperature, availability of feed and water, and to remove mortality. The BW of mortalities was used to adjust FCR. At 32 d of age, 10 birds per pen were randomly selected, wing banded, and marked on the feathers on the middle of the back with a non-toxic blue dye (commercial food coloring mixed into water) to identify the birds to be processed on day 36. The wing bands had numbers engraved on them to identify bird number, pen, and treatment, and to calculate carcass and parts yield after processing. Wing banding was conducted 4 days before processing to reduce bird stress. Feed was withdrawn 10 h prior to processing, but birds had ad libitum access to water during the feed withdrawal period. On day 36, birds were placed in coops and transferred to the Auburn University Processing Plant. Broilers were electrically stunned, exsanguinated, scalded, picked, and eviscerated mechanically. The carcasses were chilled in an ice bath at a temperature of ≤4 °C for 4 h, and hanged on a rack for 5 min for draining before the chilled carcass weights were determined. The next day, carcasses were deboned by professional deboners from a commercial processing plant using stationary cones, following procedures adapted from Smith [28]. Wings were obtained by performing a cut at the shoulder joint (proximal humerus end). Leg quarters were obtained by performing two cuts, one at the joint between the femur and ilium, and one at the joint between the metatarsus and tibia at the hock joint. Skinless whole breast was obtained by cutting the ribs and separating the whole breast from the back frame, and removing the skin manually. After that, the whole breast was separated into breast fillets (pectoralis major) and tenders (pectoralis minor). The weight of skinless breast fillets, tenders, wings, and leg quarters was obtained to calculate part yields. The carcass yield was calculated relative to 36 d of age live weight, while breast, tender, wing, and leg yield were calculated relative to the chilled carcass weight.

### 2.5. Nutrient Digestibility Analysis

An indigestible marker (0.5% titanium dioxide (TiO_2_)) was added during the finisher (21 to 35 d of age) experimental diets to assess the apparent ileal digestibility (AID) of nutrients. On day 35, 4 birds per pen were randomly selected and euthanized by CO_2_ asphyxiation and cervical dislocation to collect ileal digesta and evaluate digestibility of CP, fat, and minerals. Ileal digesta was collected from 2 cm posterior of Meckel’s diverticulum to the 2 cm anterior from the ileal–cecal junction by gently squeezing out the ileal contents to provide sufficient sample (>10 g freeze dried) for nutrient digestibility analysis. While squeezing out the ileal content, distilled water was used to aid in the removal of digesta. Ileal contents were pooled within individual pens. After collection, pooled ileal samples were stored on ice, transported to the laboratory, and frozen at −20 °C until analysis. Ileal digesta were lyophilized in a Virtis Genesis Pilot Lyophilizer (SP Industries, Warminster, PA, USA) and then ground in an electric coffee grinder (Capresso 560.4 Infinity, Montvale, NJ, USA) on the finest setting. Dried ground ileal digesta and feed samples were sent to a commercial laboratory (Dairy One Forage Laboratory, Ithaca, NY, USA) to be analyzed for CP (AOAC International [25] Official Method 990.03), ether extract (AOCS [29] Standard Procedure Am 5-04), and minerals (Wolf et al. [30] and CEM Application Notes for Acid Digestion). The titanium dioxide concentration of feed and ileal digesta was determined through procedures established by Short et al. [31]. Apparent ileal digestibility of CP, fat, and minerals was calculated based on procedures from Stein et al. [32].

### 2.6. Statistical Analysis

Nutritional characterization and particle size of corn data were analyzed as a one-way ANOVA, using the GLM procedure from the JMP software [33]. Growth performance, carcass traits and nutrient digestibility data were analyzed using a randomized complete-block design, with pen location considered as the blocking factor. Each treatment had 12 replications, with pen being the experimental unit. Data were analyzed as a one-way ANOVA, using the GLM procedure from the JMP software [33]. The least-square means among the treatments were compared using Tukey’s HSD procedure, with statistical significance considered at *p* ≤ 0.05.

## 3. Results

### 3.1. Physical Characteristics

The physical characteristics of corn of different origins can be observed in Table 2. Test weight (≤0.50 lb/bu) and damaged kernels (≤1.30%) had a low variability among the corn samples of different origins. However, the % of BCFM appeared 6.0 and 6.9% higher for corn from the USA (8.20%) in comparison with corn from BRA (2.20%) and ARG (1.30%), respectively.

Particle size of corn of different origins can be observed in Table 3. Geometric mean diameter of particles of the three different types of corn averaged at 540 μm. The geometric mean diameter of corn from BRA (570 μm) was coarser (*p* = 0.013) than the particle size of the corn from the USA (500 μm); however, it was not different (*p* > 0.05) from the particle size of the corn from ARG (549 μm).

### 3.2. Proximal Analysis

Nutritional composition of the corn samples of different origins can be observed in Table 4. Moisture variation among the corn samples was ≤0.92% and all samples were close to 12%, which was suitable for the manufacture of the experimental diets. No differences (*p* > 0.05) were observed in CP among the corn samples. However, corn from ARG (8.59%) had a 0.27 and 0.33% higher CP compared than corn from BRA (8.32%) and the USA (8.26%), respectively. Similarly, no differences (*p* > 0.05) were observed for neutral detergent-insoluble CP, soluble CP, and adjusted CP among corn origins.

Corn from the USA (72.05%) had a higher (*p* < 0.001) starch content than corn from ARG (70.93%), which was higher (*p* < 0.001) than the starch content of corn from BRA (70.05%). In contrast, corn from BRA (4.58 and 4.18%) had a higher (*p* < 0.001) content of both fat and total fatty acids compared with corn from ARG (4.15 and 3.94%), which had higher contents (*p* < 0.001) than corn from the USA (3.92 and 3.76%).

Within the mineral fraction of the kernels, no differences (*p* > 0.05) were observed in the content of magnesium (Mg) and potassium (K) of the corn samples. However, corn from BRA (0.03%) had a higher (*p* < 0.001) content of calcium (Ca) in comparison with corn from ARG (0.02%) and the USA (0.02%), whereas corn from the USA (0.30%) and ARG (0.30%) had a higher (*p* = 0.004) phosphorus (P) content compared with corn from BRA (0.28%).

### 3.3. Amino Acid Content

Amino acid content of the corn samples of different origins can be observed in Table 5. Amino acids of importance for broiler production, such as methionine (Met) plus cysteine (0.02%), lysine (Lys) (0.01%), threonine (0.01%), valine (0.02%), and isoleucine, (0.02%) had very low variability (dry matter) among corn samples.

### 3.4. Broiler Performance

Broiler performance results can be observed in Table 6. No statistical differences (*p* > 0.05) were observed in BW throughout the experimental period. Nonetheless, 35-day old broilers fed diets containing corn from BRA (2608 g/bird) had the highest BW and were 1.3% heavier than the birds fed diets containing corn from the USA (2574 g/bird), and 2.6% heavier than the birds fed diets containing corn from ARG (2540 g/bird). Likewise, no statistical differences (*p* > 0.05) were observed in BWG throughout the experimental period. However, broilers fed diets containing corn from BRA (2564 g/bird) exhibited the highest BWG from 1 to 35 d of age, which was 1.3% higher than broilers fed diets with the inclusion of corn from the USA (2530 g/bird), and 2.7% higher than broilers fed diets with the inclusion of corn from ARG (2495 g/bird).

Broilers fed diets with the inclusion of corn from ARG (304 g/bird) had a higher (*p* = 0.011) FI from 1 to 10 d of age compared with broilers fed diets containing corn from the USA (294 g/bird), but was similar (*p* > 0.05) to the FI of broilers fed diets with inclusion of corn from BRA (300 g/bird). No differences (*p* > 0.05) were observed in FI among treatments from 1 to 21 and 1 to 35 d of age.

Feed conversion ratio from 1 to 10 d of age was higher (*p* = 0.019) for broilers fed diets with corn from ARG (0.966 g:g) compared with broilers fed diets containing corn from the USA (0.938 g:g), but not different from broilers fed diets containing corn from BRA (0.948 g:g).

A similar response was observed in FCR throughout the rest of the experimental period, as broilers subjected to diets with ARG corn inclusion exhibited a higher FCR than broilers fed diets with corn from BRA and the USA, from both 1 to 21 (1.248 vs. 1.226 and 1.215; *p* < 0.0001) and 1 to 35 (1.452 vs. 1.434 and 1.434; *p* < 0.002) d of age.

### 3.5. Processing Yield

Processing characteristics of broilers reared up to 35 d of age can be observed in Table 7. Overall, the inclusion of corn with different origins did not influence (*p* > 0.05) processing characteristics, except for breast fillet weight, which was higher (*p* = 0.036) for broilers fed diets with the inclusion of corn from BRA (575 g/bird) compared with broilers fed diets with corn form ARG (553 g/bird), but not different (*p* > 0.05) from the breast fillet weight of broilers fed diets with addition of corn from the USA (556 g/bird).

### 3.6. Nutrient Digestibility

Nutrient digestibility results of broilers from 21 to 35 d of age can be observed in Table 8. The inclusion of corn of different origins did not influence (*p* > 0.05) CP and crude fat digestibility among the experimental treatments. However, the AID of P (63.37 and 62.23 vs. 55.26%; *p* = 0.0003), Ca (41.59 and 43.85 vs. 30.23%; *p* = 0.0003), and K (88.98 and 87.97 vs. 86.04%; *p* = 0.001) was higher in broilers fed diets containing corn from the USA and BRA in comparison with broilers fed diets containing corn from ARG.

## 4. Discussion

Differences in physical and chemical characteristics of corn associated with its origin have an impact on its nutritional value, which consequently influenced the growth performance, carcass traits, and nutrient digestibility of broilers in the present study. Variation in the % of BCFM among corn samples might have been caused by the high content of vitreous endosperm in South American varieties (flint endosperm cultivars) [17], which produces kernels with higher hardness values [34]. In contrast, the high content of floury endosperm of varieties in the USA (dent endosperm cultivars) [35] produces kernels with a lower hardness [34]. Differences in total stress cracks between the corn samples could be partially explained by the higher susceptibility to stress cracking of kernels with a higher % of vitreous endosperm [36], characteristic of varieties in South America. However, this observation does not align with the low % of total stress cracks reported for corn from BRA; therefore, it is possible that the observed differences might have been influenced by the differences in drying temperature as well [36]. The difference in particle size of ≤70 μm between corn samples probably had no impact on broiler performance and nutrient utilization, as previously reported by Chewning et al. [37], who did not find differences (*p* > 0.05) in the FCR from 1 to 44 d of age and BW at 44 d of age of broilers fed pelleted diets with an inclusion of corn ground to either 300 or 600 μm.

Variability in the contents of starch and fat could be attributable to morphological kernel differences due to different genetic backgrounds, such as variations in the proportion between the germ and endosperm, which are the main kernel storage sites for oil and starch, respectively [7,38]. Within the mineral fraction, variability in the content of Ca and non-phytate P between corn samples could be considered negligible, since corn naturally contributes with minimum quantities of these minerals to poultry diets [39].

The growth performance results of the present study were similar to the findings by Melo-Durán et al. [40], who subjected broilers from 1 to 42 d of age to corn–soybean meal-based diets, with an inclusion of a fixed amount of corn from eight different varieties. These authors reported no significant differences (*p* > 0.05) in BW throughout the experiment. Nonetheless, these authors reported a trend (*p* = 0.096), as birds fed diets with the inclusion of the corn variety with the highest oil content and highest gross energy were among the heaviest in the experiment by its conclusion. Likewise, Giacobbo et al. [41] found no significant differences (*p* > 0.05) in the main effect of the inclusion of three different corn hybrids in diet on BWG and FCR of broilers from 1 to 42 d of age; however, this observed similar growth performance could be attributed to a similar proximate composition of the corn hybrids, as the variation in fat content was <0.11% and CP < 0.45%. On the contrary, Zhao et al. [42] reported a better performance in terms of BWG and FCR of broilers from 1 to 42 d of age fed corn–soybean meal-based diets with the inclusion of flint corn in comparison to broilers fed diets with dent corn inclusion. These authors attributed these responses to a possible better protein synthesis of young broilers fed flint corn based on lower uric acid levels found in their plasma, in comparison to young broilers fed dent corn. Differences in FCR during the starter period are in close agreement with those report by Moore et al. [39], where the feed utilization of broilers from 0 to 14 d of age was negatively correlated to the content of ADF in six corn hybrids included at the same amount to the corn–soybean meal-based starter diets. A possible explanation for this observation might be the encapsulation of starch granules by fiber, which restricts the access of digestive enzymes to the starch granules [39]. In the current study, birds fed diets with ARG corn inclusion had a lower BW at 35 d of age, lower BWG from 1 to 35 d of age, and higher FCR from 1 to 35 d of age in comparison to the rest of the treatments. Corn from ARG had the highest content of CP among all corn samples, indicating a higher amount of zeins [43], which are storage structures that have been reported to influence the texture of the kernel [44]. Kljak et al. [45] reported an increase in the vitreousness of kernels with an increase in the content of zeins. More vitreous grains may negatively influence the performance of broilers through lower protein and starch digestibility caused by restriction of the access of endogenous enzymes to these nutrients [5]. In previous research, Jaeger et al. [46] found a decrease in feed efficiency of cattle fed corn hybrids with a harder endosperm.

Overall, research by Moore et al. [39] describes variability in physical characteristics and chemical composition of corn as the most impactful factors of nutrient utilization for young broilers with an underdeveloped gastrointestinal tract, and older broilers with a mature gastrointestinal tract, respectively.

In the present experiment, differences in breast weight fillets could not be attributed to differences in the concentration of Lys and Met of the corn samples, since a very low variability was found for these AAs. Alternatively, differences in energy availability resulting from physical and chemical characteristics could have been more impactful in feed utilization and growth of the birds than AA concentration of the samples, as reported by Moore et al. [39]. Similarly, Zhao et al. [47] found no differences (*p* > 0.05) in the whole breast weight of broilers fed a corn–soybean meal-based diet with the inclusion of either dent or flint corn at fixed amounts, even though they had differences of 0.18 and 0.16% within their contents of Lys and Met, respectively. Overall, similar results were obtained by Giacobbo et al. [41], who did not report differences (*p* > 0.05) in carcass, whole breast, wing, and leg yield of 42-day old broilers subjected to diets with the inclusion of three different corn varieties.

Crude fat digestibility values displayed by broilers in the present study are close to values within the report by Kaczmarek et al. [48], who reported a crude fat digestibility of 86.7 and 87.9% by 5-week old broilers fed a corn–soybean meal-based diet, with 50% inclusion of corn with and without the inclusion of exogenous enzymes, respectively. Likewise, the average AID for P (60.3%) in the present experiment is close to that report by Cowieson et al. [49], who demonstrated a retention of 64% of P by female broiler chickens fed a diet with 56% inclusion of corn and 300 FTU/kg of phytase. According to Eeckhout and De Paepe [50], the availability of P in ingredients used for animal nutrition depends on the content of phytate P, non-phytate P, and phytase activity of the diet. Therefore, a possible explanation for differences observed in the AID of P could be the higher content of phytate P and lower content of non-phytate P in corn from ARG, caused by a different genetic background [51]. Calcium digestibility of broilers fed diets containing corn form ARG was likely affected, as more Ca might have been bound to the phytate complex, limiting its absorption by the birds [51]. The high inclusion of corn in broiler diets, plus its high concentration of K (0.28–0.33% as-fed basis) [43,52], could make corn a considerable contributor to this mineral in broiler diets. Potassium is an intracellular cation with direct influence on the cation–anion balance of organisms [53], and has direct influence on the of thermotolerance capacity of broilers [54]. Supplementation of K in broiler diets has been previously associated with an increase in BWG in heat-stressed birds [54,55,56]. Therefore, a higher digestibility of K by broilers fed diets with inclusion of corn from the USA and BRA in comparison to broilers fed diets with the inclusion of corn from ARG, might have had a partial contribution to their better performance when compared with broilers fed corn from ARG. The underlying mechanism responsible for the observed differences in K digestibility is unclear; however, it might be related with its interactions with dietary Ca concentration, as reported by a recent report by Bradbury et al. [57].

## 5. Conclusions

In conclusion, male YPM × Ross 708 broilers fed diets with the inclusion of corn from the USA and BRA exhibited an improvement in FCR from 1 to 21 and 1 to 35 d of age, in comparison to broilers fed diets with the inclusion of corn from ARG. For processing characteristics, corn origin only influenced breast meat weight, which was higher for broilers fed diets with the inclusion of corn from BRA in comparison to broilers fed diets with inclusion of corn from ARG, but was not different from the breast weight of broilers fed diets with the inclusion of corn from the USA. Inclusion of corn with different origins did not influence CP and crude fat digestibility; however, broilers fed diets with the inclusion of corn from the USA and BRA exhibited greater P, Ca, and K digestibility from 21 to 35 d of age. In addition, from a feed milling perspective, the findings of this study highlight the need to understand the nutritional composition of corn batches prior the formulation of broiler feeds, and provides evidence that corn origin influences its nutrient content and digestibility, as well as broiler performance. The results of this trial could be used by poultry nutritionists to make decisions between feed formulation cost and economic returns from broiler performance.

## Figures and Tables

**Table 1:** Ingredient and nutrient composition (% as-fed basis, unless otherwise indicated) of dietary treatments fed to YPM × Ross 708 male broilers from 1 to 35 d of age.

Ingredient	Starter 1 to 10 d	Grower 11 to 21 d	Finisher 21 to 35 d
Corn	53.81	58.04	61.76
Soybean meal, 46.9% CP	34.92	30.22	25.52
DDGS	5.00	5.00	5.00
Poultry oil	2.77	3.63	4.49
Limestone	1.13	1.05	0.98
Dicalcium phosphate, 18% P	0.98	0.76	0.55
Salt	0.43	0.43	0.43
DL-Methionine, 99%	0.33	0.30	0.26
L-Lysine	0.22	0.20	0.19
L-Threonine, 98%	0.14	0.11	0.08
Choline chloride, 70%	0.08	0.08	0.08
Trace mineral premix ^1^	0.10	0.10	0.10
Vitamin premix ^2^	0.10	0.08	0.05
Titanium dioxide	0.00	0.00	0.50
OptiPhos^®^ Plus ^3^, g/kg	0.05	0.05	0.05
Calculated analysis			
AME_n_^4^, kcal/kg	3000	3100	3200
Crude protein	22.37	20.35	18.35
Digestible Lys	1.23	1.10	0.98
Digestible TSAA ^5^	0.91	0.84	0.77
Digestible Thr	0.83	0.74	0.65
Digestible Ile	0.83	0.75	0.67
Digestible Val	0.92	0.84	0.76
Digestible Arg	1.35	1.21	1.07
Calcium	0.96	0.87	0.78
Available phosphorus	0.48	0.44	0.39

^1^ Mineral premix includes per kg of diet: Mn (manganese sulfate), 120 mg; Zn (zinc sulfate), 100 mg; Fe (iron sulfate monohydrate), 30 mg; Cu (tri-basic Cu chloride), 8 mg; I (ethylenediaminedihydroxide), 1.4 mg; Se (sodium selenite), 0.3 mg. ^2^ Vitamin premix includes per kg of diet: Vitamin A (Vitamin A acetate), 187,390 IU; Vitamin D (cholecalciferol), 6614 IU; Vitamin E (DL-alpha tocopherol acetate), 66 IU; menadione (menadione sodium bisulfate complex), 4 mg; Vitamin B12 (cyanocobalamin), 0.03 mg; folacin (folic acid), 2.6 mg: D-pantothenic acid (calcium pantothenate), 31 mg; riboflavin (riboflavin), 22 mg; niacin (niacinamide), 88 mg; thiamin (thiamin mononitrate), 5.5 mg; D-biotin (biotin), 0.18 mg; and pyridoxine (pyridoxine hydrochloride), 7.7 mg. ^3^ OptiPhos^®^ Plus (Huvepharma Inc., Peachtree City, GA, USA) provided 500 FTU/kg of phytase activity per kg of diet. ^4^ Apparent metabolizable energy corrected to nitrogen equilibrium. ^5^ Total sulfur amino acids.

**Table 2:** Analyzed physical characteristics (% as-received basis, unless otherwise indicated) of corn samples of different origins used for the manufacture of the experimental dietary treatments fed to YPM × Ross 708 male broilers from 1 to 35 d of age.

Physical Characteristics	USA	ARG	BRA
Test weight, lb/bu	58.10	58.00	58.50
Damaged kernels	0.50	1.50	0.20
Broken corn and foreign material	8.20	1.30	2.20
Total stress cracks	4.00	16.00	2.00

**Table 3:** Particle size distribution (%, unless otherwise indicated) of corn samples of different origins used for the manufacture of the experimental dietary treatments fed to YPM × Ross 708 male broilers from 1 to 35 d of age ^1^.

Sieve Size, µm	USA	ARG	BRA	SEM ^2^	*p*-Value
4760	0.00	0.00	0.00	-	-
3360	0.45	0.47	0.25	-	-
2380	1.99	1.86	1.98	-	-
1680	6.58	7.09	7.28	-	-
1190	15.06	16.52	16.54	-	-
840	15.20	16.71	16.57	-	-
590	13.73	14.55	15.03	-	-
420	10.60	11.32	11.65	-	-
297	7.67	7.65	8.03	-	-
210	6.26	6.04	6.28	-	-
149	4.57	5.15	4.92	-	-
105	3.03	2.11	2.58	-	-
74	2.84	2.28	2.60	-	-
53	2.22	1.90	2.19	-	-
37	9.80	6.35	4.10	-	-
Gdw ^3^, μm	500 ^b^	549 ^ab^	570 ^a^	14	0.013

^a,b^ Least-square means within a row with different superscripts differ significantly (*p* ≤ 0.05). ^1^ Analysis was performed on five replicates. ^2^ Standard error of the mean. ^3^ Geometric mean diameter.

**Table 4:** Analyzed chemical composition (% as-fed basis, unless otherwise indicated) of corn samples of different origins used for the manufacture of the experimental dietary treatments fed to YPM × Ross 708 male broilers from 1 to 35 d of age ^1^.

Nutrient Composition	USA	ARG	BRA	SEM ^2^	*p*-Value
Moisture	12.97 ^a^	12.82 ^a^	12.05 ^b^	0.09	<0.001
Dry matter	87.03 ^b^	87.18 ^b^	87.95 ^a^	0.09	<0.001
Crude protein (CP)	8.26	8.59	8.32	0.12	0.150
Acid detergent-insoluble CP	0.60 ^b^	0.64 ^a^	0.66 ^a^	0.01	0.002
Neutral detergent-insoluble CP	1.49	1.55	1.50	0.02	0.152
Soluble protein, % CP	26.64	26.61	28.78	0.83	0.147
Acid detergent fiber	2.38 ^b^	2.79 ^a^	2.82 ^a^	0.10	0.020
Neutral detergent fiber	7.53 ^b^	8.38 ^a^	8.41 ^a^	0.16	0.003
Water-soluble carbohydrates	2.03	2.10	2.12	0.07	0.672
Ethanol-soluble carbohydrates	1.59	1.64	1.65	0.69	0.056
Starch	72.05 ^a^	70.93 ^b^	70.05 ^c^	0.23	<0.001
Fat, ether extract	3.92 ^c^	4.15 ^b^	4.58 ^a^	0.05	<0.001
Total fatty acids (TFA)	3.76 ^c^	3.94 ^b^	4.18 ^a^	0.03	<0.001
Ash	1.45	1.45	1.47	0.03	0.842
Calcium	0.02 ^b^	0.02 ^b^	0.03 ^a^	0.001	<0.001
Phosphorus	0.30 ^a^	0.30 ^a^	0.28 ^b^	0.004	0.004
Magnesium	0.12	0.12	0.11	0.002	0.033
Potassium	0.38	0.38	0.37	0.01	0.220
Adjusted CP	8.26	8.59	8.32	0.12	0.150
Non-fibrous carbohydrates	79.67 ^a^	78.43 ^b^	78.41 ^b^	0.22	0.002
Palmitic acid	0.49 ^c^	0.51 ^b^	0.54 ^a^	0.004	<0.001
Stearic acid	0.06 ^b^	0.06 ^b^	0.08 ^a^	0.002	<0.001
Oleic acid	1.06 ^c^	1.11 ^b^	1.17 ^a^	0.01	<0.001
Linoleic acid	2.03 ^c^	2.13 ^b^	2.25 ^a^	0.02	<0.001
Linolenic acid	0.05 ^b^	0.06 ^ab^	0.062 ^a^	0.002	0.024
Palmitic acid, %TFA	13.08 ^a^	12.98 ^ab^	12.91 ^b^	0.03	0.014
Stearic acid, %TFA	1.54 ^b^	1.52 ^b^	1.814 ^a^	0.04	0.001
Oleic acid, %TFA	28.23	28.25	28.06	0.07	0.176
Linoleic acid, %TFA	53.85	53.95	53.83	0.09	0.612
Linolenic acid, %TFA	1.43	1.52	1.48	0.04	0.344

^a–c^ Least-square means within a row with different superscripts differ significantly (*p* ≤ 0.05). ^1^ Least-square means of five replicates. ^2^ Standard error of the mean.

**Table 5:** Analyzed amino acid content (% dry matter basis) of corn samples of different origins used for the manufacture of the experimental dietary treatments fed to YPM × Ross 708 male broilers from 1 to 35 d of age.

Amino Acid	USA	ARG	BRA
Alanine	0.61	0.62	0.57
Arginine	0.35	0.33	0.34
Aspartic acid	0.56	0.55	0.54
Cysteine	0.19	0.18	0.19
Glutamic acid	1.52	1.53	1.42
Glycine	0.34	0.33	0.33
Histidine	0.26	0.25	0.25
Hydroxylysine	0.00	0.00	0.00
Hydroxyproline	0.03	0.02	0.03
Isoleucine	0.33	0.33	0.31
Lanthionine ^§^	0.01	0.01	0.01
Leucine	1.00	1.01	0.92
Lysine	0.28	0.27	0.27
Methionine + cysteine	0.35	0.33	0.35
Methionine	0.16	0.15	0.17
Ornithine ^§^	0.01	0.01	0.01
Phenylalanine	0.43	0.43	0.39
Proline	0.71	0.71	0.71
Serine	0.36	0.37	0.35
Taurine ^§^	0.23	0.23	0.24
Threonine	0.29	0.30	0.29
Tryptophan	0.05	0.05	0.06
Tyrosine	0.17	0.15	0.15
Valine	0.42	0.42	0.40

^§^ Non-proteinogenic amino acids.

**Table 6:** Performance of YPM × Ross 708 male broilers fed diets with the inclusion of corn of different origins from 1 to 35 d of age.

Treatment	BW ^1^, g/Bird			BWG ^2^, G/Bird			FI ^3^, g/Bird			FCR ^4^, g:g		
	10 d	21 d	35 d	1–10 d	1–21 d	1–35 d	1–10 d	1–21 d	1–35 d	1–10 d	1–21 d	1–35 d
USA	314	1073	2574	269	1029	2530	294 ^b^	1304	3697	0.938 ^b^	1.215 ^b^	1.434 ^b^
ARG	315	1048	2540	270	1003	2495	304 ^a^	1310	3693	0.966 ^a^	1.248 ^a^	1.452 ^a^
BRA	315	1065	2608	271	1021	2564	300 ^ab^	1313	3756	0.948 ^ab^	1.226 ^b^	1.434 ^b^
SEM ^5^	3	8	21	3	8	21	2	9	27	0.007	0.003	0.004
*p*-value	0.906	0.099	0.078	0.922	0.081	0.073	0.011	0.788	0.189	0.019	<0.0001	0.002

^a,b^ Least-square means within a column with different superscripts differ significantly (*p* ≤ 0.05). ^1^ Body weight. ^2^ Body weight gain. ^3^ Feed intake. ^4^ Feed conversion ratio corrected for mortality. ^5^ Standard error of the mean.

**Table 7:** Carcass characteristics of YPM × Ross 708 male broilers fed diets with the inclusion of corn of different origins from 1 to 35 d of age.

Treatment	Chilled Carcass		Breast Fillets		Tenders		Wings		Legs	
	Weigh, g/Bird	Yield ^1^, %	Weigh, g/Bird	Yield ^2^, %	Weigh, g/Bird	Yield ^2^, %	Weigh, g/Bird	Yield ^2^, %	Weigh, g/bird	Yield ^2^, %
USA	1983	73.95	556 ^ab^	27.99	114	5.77	200	10.08	566	28.58
ARG	1990	74.10	553 ^b^	27.72	114	5.71	200	10.08	570	28.71
BRA	2029	74.23	575 ^a^	28.30	114	5.64	203	10.04	581	28.63
SEM ^3^	16.21	0.55	6.47	0.17	1.33	0.05	1.56	0.05	4.75	0.13
*p*-value	0.094	0.938	0.036	0.065	0.908	0.178	0.172	0.816	0.097	0.746

^a,b^ Least-square means within a column with different superscripts differ significantly (*p* ≤ 0.05). ^1^ Chilled carcass yield was calculated as the percentage of live weight at 36 d of age. ^2^ Yields calculated as a percentage of the carcass weight. ^3^ Standard error of the mean.

**Table 8:** Apparent ileal nutrient digestibility (%) of YPM × Ross 708 male broilers fed diets with inclusion of corn of different origins from 21 to 35 d of age.

Treatment	Apparent Ileal Digestibility				
	Crude Protein	Crude Fat	Phosphorus	Calcium	Potassium
USA	80.76	91.38	63.37 ^a^	41.59 ^a^	88.98 ^a^
ARG	79.90	91.04	55.26 ^b^	30.23 ^b^	86.04 ^b^
BRA	80.57	90.73	62.23 ^a^	43.85 ^a^	87.97 ^a^
SEM ^1^	0.50	0.63	1.37	3.18	0.74
*p*-value	0.470	0.766	0.0003	0.0003	0.001

^a,b^ Least-square means within a column with different superscripts differ significantly (*p* ≤ 0.05). ^1^ Standard error of the mean.

## Data Availability

All data are contained within the article.
